# Enhanced Properties of Alumina Cement Adhesive for Large-Tonnage Insulator Under Rapid Curing Regime

**DOI:** 10.3390/ma19010171

**Published:** 2026-01-03

**Authors:** Weibing Zhou, Yongchao Min, Jun Zhou, Shouqin Tian

**Affiliations:** 1 School of Materials Science and Engineering, Wuhan University of Technology, Wuhan 430070, China; myc@whut.edu.cn; 2 China Electric Power Research Institute Co., Ltd., Beijing 100089, China; zhouj1gw@163.com; 3 State Key Laboratory of Advanced Glass Materials, Wuhan University of Technology, Wuhan 430070, China; tiansq@whut.edu.cn

**Keywords:** alumina cement adhesive, curing regime, hydration products, large-tonnage insulator, thermomechanical performance

## Abstract

The performance of cement adhesive in large-tonnage insulators is crucial for determining their structural stability and service life when subjected to long-term electromechanical loading and complex environmental interactions. This work addresses the issue of late-stage strength reduction in alumina cement by employing a rapid steam curing process. The influence of curing temperature on the phase composition and microstructure of the hydration products is investigated, along with the evolution over time of the mechanical properties, dry shrinkage rate and elastic modulus. These findings are further validated through thermal–mechanical performance testing of bonded insulators. The results demonstrate that: (1) The hydration products of the adhesive are significantly influenced by steam curing temperature: the metastable phase CAH_10_ forms at 20 °C; it transforms into the metastable phase C_2_AH_8_ at 50–60 °C; it changes to the stable phase C_3_AH_6_ at 70 °C; and microcracks appear and porosity increases at 80–90 °C, although the stable phase C_3_AH_6_ remains the dominant phase. (2) Alumina cement adhesive prepared via 2 h steam curing at 70 °C exhibited superior properties, with flexural and compressive strengths reaching 14.2 MPa and 112.7 MPa, respectively. After 360 days, flexural strength remained above 12 MPa and compressive strength exceeded 110 MPa. Dry shrinkage was below 0.04%, with an elastic modulus of approximately 49.6 GPa. (3) Microstructural analysis revealed that the hydration products of the cured adhesive were predominantly C_3_AH_6_ and AH_3_, exhibiting stable structures. After 90 days, porosity decreased to 3.56%, with the C_3_AH_6_ and AH_3_ gels tightly enveloping the aggregates and forming a dense, three-dimensional network structure. (4) All bonded insulators successfully passed thermomechanical performance tests. Therefore, this work can provide a good way to prepare a high-performance cement adhesive for insulators.

## 1. Introduction

In the context of the ongoing construction and upgrading of ultra-high voltage transmission projects, there is an increasing demand for large-tonnage insulators. This demand is accompanied by increasingly stringent requirements for their mechanical properties and long-term reliability [1,2]. As the critical bonding material for large-tonnage glass or porcelain insulators, the performance of cement adhesive directly determines the structural stability and service life of insulators under long-term exposure to electromechanical loads and complex environmental conditions [3,4,5].

Currently, the manufacture of large-tonnage domestic insulators predominantly employs Portland cement adhesive [6,7,8]. However, issues with these adhesives include low early strength, prolonged curing times and high drying shrinkage [9,10]. These limitations constrain production efficiency and pose risks of microcracking or detachment during long-term operation, which threatens the safety and stability of power grids. In contrast, alumina cement offers advantages such as high early strength, low drying shrinkage, minimal porosity and short curing times [11,12], which significantly enhance the production efficiency and properties stability of insulators. In the ultra-high voltage (UHV) engineering sector, Japan’s NGK Insulators has successfully used alumina cement bonding agents for large-tonnage porcelain insulators. However, domestic enterprises have yet to achieve the large-scale application and promotion of this product. The main obstacle is the lack of systematic research by domestic enterprises and scholars into the properties of alumina cement. This prevents a fundamental solution to the potential reduction in strength in the later stages, which occurs when the metastable phases CAH_10_ and C_2_AH_8_ transform into the stable phase C_3_AH_6_. This leads to increased porosity, volume changes and a reduction in strength [13,14].

Researchers have conducted long-term, systematic studies on the hydration products in alumina cement mortars, investigating their impact on properties [15,16,17]. These studies indicate that the properties of alumina cement are significantly influenced by curing conditions. For example, Kula et al. [15] systematically investigated the impact of different curing temperatures and humidity levels on the strength and phase composition of alumina cement paste. They discovered that, under 60 °C curing conditions, the primary hydration products were C_3_AH_6_ and AH_3_, which exhibited significantly greater strength than the CAH_10_ system formed under 21 °C curing conditions. Furthermore, this transformation process did not result in strength deterioration. K. Scrivener et al. [16] systematically elucidated the phase transformation process of calcium aluminate cement (CAC) in 2003. Ahmed et al. [17] revealed the effects of curing temperature and water-to-cement ratio on alumina cement concrete. They confirmed that high-temperature curing at 90 °C promotes the formation of C_3_AH_6_. P. Garcés et al. [18] demonstrated, through kinetic studies, that, at a curing temperature of 60 °C, the metastable phases CAH_10_ and C_2_AH_8_ were scarcely detectable in the hydration products. Instead, stable coexisting structures of C_3_AH_6_ and AH_3_ were formed. Nowacka et al. [19] systematically elucidated the control that temperature exerts over hydration pathways. At a low-temperature curing of 6 °C, CAH_10_ predominates. When the temperature rises to 25–30 °C, CAH_10_ and C_2_AH_8_ coexist and gradually become dominant. In contrast, at curing temperatures of 40 °C or 60 °C, a stable system is formed directly that is dominated by C_3_AH_6_ and AH_3_. Goergens et al. [20] conducted dynamic observations using in situ X-ray diffraction and confirmed that, when curing temperatures exceed 40 °C, the hydration reaction pathway undergoes a fundamental shift. Nucleation and growth of metastable phases are strongly suppressed, and the hydration reaction is directed towards the rapid and direct formation of the stable phases C_3_AH_6_ and AH_3_.

Recent research on optimising CAC systems has focused on enhancing performance through material design and curing control. For example, addressing strength degradation caused by phase transformations during high-temperature curing, Win et al. [21] investigated the positive effects of fibre reinforcement. Wang et al. [22] discovered that incorporating carbonated magnesium slag promotes the formation of C_3_A·CaCO_3_·11H_2_O, effectively suppressing the conversion of metastable phases CAH_10_ and C_2_AH_8_ and enhancing long-term strength stability. Wan et al. [23] investigated silica fume-modified alumina cement adhesives combined with hot water curing, focusing on electrical insulation properties. However, systematic analysis of key characteristics such as phase composition and drying shrinkage remains insufficient. The above studies indicate that rational material design and curing control can optimise the performance of CAC systems to a certain extent. However, systematic research on alumina cement adhesives for large-scale insulators remains relatively scarce.

This study focused on designing and preparing an alumina cement adhesive based on research into the impact of temperature on the hydration products of alumina cement. The adhesive was made using alumina cement as the cementitious material, artificial porcelain sand as the aggregate and silica fume as the mineral admixture, a rapid curing regimen was employed. This study systematically examined the flexural-compressive strength, dry shrinkage rate, elastic modulus and microstructure, through thermomechanical performance testing, the feasibility of using this cement adhesive for bonding insulators was verified, providing the necessary experimental data and theoretical basis for its application in large-tonnage insulators.

## 2. Materials and Methods

### 2.1. Materials

**Figure 1:**
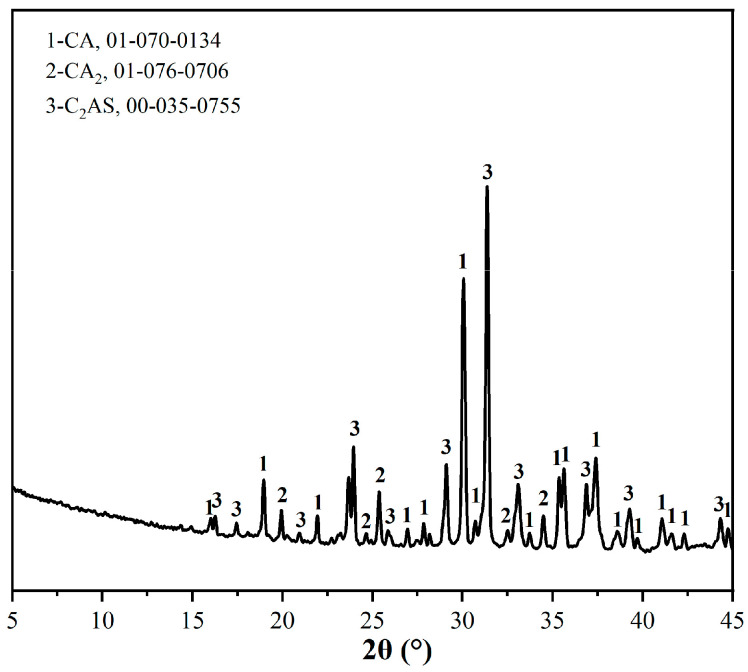
XRD patterns of alumina cement.

CA 50 alumina cement, produced by China Great Wall Aluminum Cement Group Co., Ltd. (Zhengzhou, China), was used. Its chemical composition was independently determined using a Zetium X-ray fluorescence spectrometer (Malvern Paralytical, Shanghai, China), as shown in Table 1. and its mineral composition was depicted in Figure 1. It primarily consisted of calcium aluminate (CA), calcium dialuminate (CA_2_), and calcium alumina feldspar (C_2_AS). The self-produced artificial porcelain sand aggregate was made from bauxite (Lingshou County Yanyue Mineral Products Co., Ltd., Shijiazhuang, China), kaolin (Lingshou Boneng New Materials Co., Ltd., Shijiazhuang, China), and quartz (Tuoyi New Materials Co., Ltd., Guangzhou, China) as raw materials. It was processed through wet ball milling (Wuxi Xinyang Equipment Technology Co., Ltd., Wuxi, China), spray granulation (Changzhou Jinwang Drying Equipment Co., Ltd., Changzhou, China), dry pressing (Guangzhou Jianhua Precision Technology Co., Ltd., Guangzhou, China) into bricks, and then sintered at 1600 °C. Afterwards, it was crushed and sieved to 30–70 mesh. Its main mineral components were mullite, alumina, and quartz. The silica fume, with a SiO_2_ content of 99%, was manufactured by Elkem Organic Silicon Co., Ltd., Shanghai, China. The water-reducing agent used was a high-properties polycarboxylic acid water-reducing agent, achieving a water reduction rate of 35%.

### 2.2. Sample Preparation

Alumina cement was used as the primary cementitious material in this study. Cement paste specimens were prepared using two mix designs with the following proportions: Group X1 with a water-to-binder ratio of 0.36 and Group X2 with a water-reducing agent content of 0.2% and a water-to-binder ratio of 0.18. The thoroughly mixed cement paste was poured into 40 mm in side cubic moulds and immediately placed in a steam curing chamber (Hebei Guanghui Testing Instruments Co., Ltd., Cangzhou, China) at temperatures of 20 °C, 50 °C, 60 °C, 70 °C, 80 °C and 90 °C for 2 h, labelled as T20, T50, T60, T70, T80 and T90, respectively.

Based on this, an alumina cement adhesive formulation was designed with a cement-to-aggregate ratio of 3:1, a water-to-cement ratio of 0.18, a superplasticiser content of 0.2% by cement mass and a silica fume content of 3.5% by cement mass, the prepared alumina cement adhesive exhibited an initial setting time exceeding one hour and a flow value of 300 mm. The thoroughly mixed cement adhesive mortar was then poured into strength moulds (40 mm × 40 mm × 160 mm), dry shrinkage moulds (25 mm × 25 mm × 280 mm) and elastic modulus moulds (100 mm × 100 mm × 400 mm), after pouring, the specimens were immediately transferred into a sealed steam curing chamber preheated and stabilised at 70 °C with a relative humidity of ≥95%, and cured at a constant temperature for 2 h, then cooled to room temperature (25 °C). After demoulding, the specimens were cured at room temperature until the specified age, after which properties testing was conducted.

### 2.3. Testing and Characterisation Methods

#### 2.3.1. Flexural and Compressive Strength

The strength of specimens at specified ages was determined using a HYZ-300 fully automatic flexural and compressive testing machine (Zhejiang Huanan Instrument Equipment Co., Ltd., Shaoxing, China), in accordance with the standard JB/T 4307-2004 [24]. The loading rate for the flexural strength test was set at 50 ± 5 N/s with 3 replicate specimens, while for the compressive strength test, it was set at 2.4 ± 0.5 kN/s with 6 replicate specimens.

#### 2.3.2. Drying Shrinkage

The length variation in specimens at different ages was measured using a BC156-300 comparator (Wuxi Jianyi Instrument Machinery Co., Ltd., Wuxi, China). For each condition, 3 parallel specimens were measured, and the drying shrinkage was taken as the average of the 3 measurements. The drying shrinkage (*S_t_*, %) was calculated using Equation (1):

(1) $$S_{t}=\frac{L_{0}-L_{1}}{L}\times 100\%$$

where

*S_t_* is the drying shrinkage, %;

*L*_0_ is the initial length, mm;

*L*_1_ is the length at the specified age, mm;

*L* is the effective length of the specimen (the fixed distance between the measuring gauge points, 250 mm).

#### 2.3.3. Dynamic Modulus of Elasticity

The dynamic modulus of elasticity of specimens at different ages was measured using a DT-20W dynamic modulus tester (Shanghai Precision Instrument and Meter Co., Ltd., Shanghai, China), with a sample size of 3. The dynamic modulus of elasticity (*E*, GPa) was calculated using Equation (2):

(2) $$E=0.9465\frac{mf_{f}^{2}}{b}{\left(\frac{L}{t}\right)}^{3}\left[1+6.585{\left(\frac{t}{L}\right)}^{2}\right]$$

where

*E* is the dynamic modulus of elasticity, GPa;

*m* is the mass, kg;

*f*_f_ is the fundamental resonant frequency, Hz;

*L*, *b*, and *t* are the length (400 mm), width (100 mm), and thickness (100 mm) of the specimen, respectively.

#### 2.3.4. X-Ray Diffraction (XRD) Analysis

Phase analysis was conducted using a D8-Advance X-ray diffractometer (Bruker, Walzbach, Germany) with a Cu Kα radiation source (λ = 0.15418 nm), the diffraction patterns were recorded in continuous scan mode over a 2θ range of 5° to 70° at a scan rate of 5°/min. For XRD sample preparation, fragments from the central part of the specimens after compressive strength testing were immersed in absolute ethanol to stop hydration. Prior to testing, the samples were taken out, ground, and passed through a 200-mesh sieve to obtain the powder for analysis.

#### 2.3.5. Low-Field Nuclear Magnetic Resonance (LF-NMR) Testing

The pore structure parameters, including total porosity and pore size distribution, were determined by LF-NMR, the tests were performed using an MR12-060H-1 NMR analyzer (Niumag Analytical Instrument Co., Ltd., Suzhou, China). The resonance frequency was 23.04 MHz, the magnet temperature was controlled at 32 ± 0.01 °C, and the probe diameter was 25 mm. Transverse relaxation time (T2) measurements were used to characterise the pore characteristics of the cement adhesive specimens at 11 days of age. Before testing, the specimens were subjected to vacuum-pressure water saturation for 12 h to ensure water-filled pores. The test specimens were cube-shaped with a side length of 2 mm, and three parallel samples (n = 3) were prepared for each set of conditions. The final pore characteristic data represented the average of the three tests.

#### 2.3.6. Scanning Electron Microscopy (SEM)

The microstructure of the samples was observed using a Zeiss (Jena, Germany) Ultra Plus field emission scanning electron microscope, under an acceleration voltage of 5 kV and in secondary electron (SE2) imaging mode. The sample preparation procedure was as follows: samples were removed from absolute ethanol and dried in a vacuum oven (Guangdong Honggaoxin Testing Equipment Co., Ltd., Dongguan, China) at 20 °C for 24 h. Fragments of approximately 3 mm in size were selected, mounted on conductive adhesive, and the observation surface was coated with a platinum layer.

#### 2.3.7. Thermomechanical Performance Testing

The thermomechanical performance of the insulator porcelain bodies was tested using a QH-CHWLWL-2000K1L 2000 kN horizontal single-station thermal testing machine (Zibo Qianheng Automation Engineering Co., Ltd., Zibo, China) and a WEW-2000 universal testing machine (Jinan Times Shijin Testing Instrument Co., Ltd., Jinan, China), following the standard GB/T 1001.1-2021 [25]. The test procedure consisted of 12 thermal cycles (over 4 days). Each cycle sequentially involved: cooling to –40 °C and holding for 4 h, followed by heating to +60 °C and holding for 4 h. A tensile load equivalent to either 80% or 100% of the specified electromechanical failing load was continuously applied throughout the entire cycling process. If the sample remained undamaged, its electromechanical failing load was determined after the cycling. During this final failing load test, a 50 kV power frequency voltage was applied and maintained across each insulator unit. Simultaneously, a tensile load was applied between the metal fittings. This load was increased steadily and rapidly from zero to 80% or 100% of the specified electromechanical failing load, and then further increased at a rate ranging from 100% to 35% of the specified failing load per minute until failure of the porcelain body occurred. The maximum load sustained was recorded as the electromechanical failing load.

## 3. Results and Discussion

### 3.1. Effect of Steam Curing Temperature on Hydration Products of Alumina Cement

**Figure 2:**
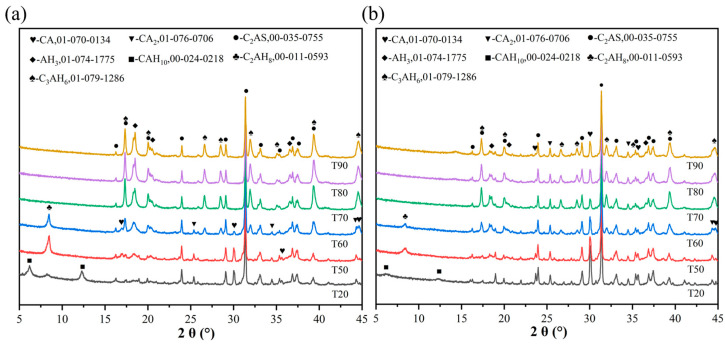
XRD patterns of alumina cement at different curing temperatures: (**a**) X1; (**b**) X2.

Figure 2 shows the XRD patterns of groups X1 and X2 under different steam curing temperatures (20, 50, 60, 70, 80, and 90 °C), revealing significant changes in the hydration products of alumina cement with varying temperatures, irrespective of the water-cement ratio. At 20 °C, distinct CAH_10_ diffraction peaks were observed without detectable C_3_AH_6_ or AH_3_ phases, indicating the formation of metastable CAH_10_ as the primary hydration product, while the high intensity of residual CA peaks suggested a low hydration degree. When the temperature increased to 50 °C, the CAH_10_ peaks disappeared, and C_2_AH_8_ and AH_3_ peaks emerged, demonstrating the conversion of hydration products to C_2_AH_8_ and AH_3_, as represented by Equation (3). At 60 °C, C_2_AH_8_ persisted while C_3_AH_6_ appeared alongside intensified AH_3_ peaks, reflecting the partial transformation of C_2_AH_8_ into C_3_AH_6_ and AH_3_, as represented by Equation (4). By 70 °C, C_2_AH_8_ peaks vanished with further intensified C_3_AH_6_ and AH_3_ peaks, confirming complete conversion to these stable phases. Concurrently, the decreasing CA peak intensity up to 70 °C indicated progressively enhanced hydration. However, at 80 °C and 90 °C, despite unchanged phase types, the increased CA peak intensity suggested reduced hydration, likely due to rapid paste hardening forming a dense layer that encapsulated unhydrated CA particles, thereby hindering water contact and further reaction [16,26].

3CAH_10_ → C_3_AH_6_+2AH_3_+18H (3)

3C_2_AH_8_ → 2C_3_AH_6_+AH_3_+9H (4)

**Figure 3:**
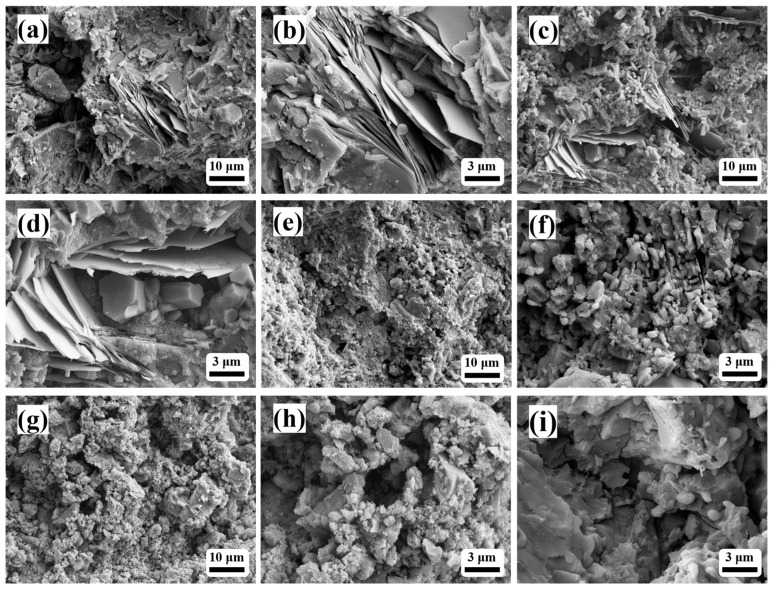
SEM images of hydration products of alumina cement at 24 h under different curing temperatures: (**a**,**b**) 20 °C; (**c**,**d**) 50 °C; (**e**,**f**) 70 °C; (**g**–**i**) 90 °C.

Figure 3 shows the microstructure of hydration products in alumina cement at 24 h under different steam curing temperatures (20, 50, 70, and 90 °C). Under the 20 °C curing condition, the hydration products were predominantly interwoven plate-like CAH_10_ crystals, forming a porous network structure with large voids, indicating slow hydration reactions and loose packing of hydrates. When the temperature increased to 50 °C, the plate-like morphology persisted, but the crystal size significantly decreased, accompanied by the appearance of fine granular phases and reduced porosity, suggesting accelerated hydration and denser packing. Concurrently, part of the CAH_10_ transformed into smaller hexagonal plate-like C_2_AH_8_ crystals, consistent with the XRD results shown in Figure 2. At 70 °C, plate-like crystals substantially diminished, replaced by abundant fine granular C_3_AH_6_, resulting in a markedly densified microstructure with smaller pores. When the temperature reached 90 °C, plate-like crystals almost completely disappeared, and the hydration products mainly consisted of fine granular agglomerates, presenting a compact structure. However, microcracks were observed, likely originating from volumetric shrinkage induced by rapid hydration at elevated temperatures. Although complete conversion to the stable C_3_AH_6_ phase was achieved, such structural defects could impair the mechanical properties of the cementitious matrix [27,28].

**Figure 4:**
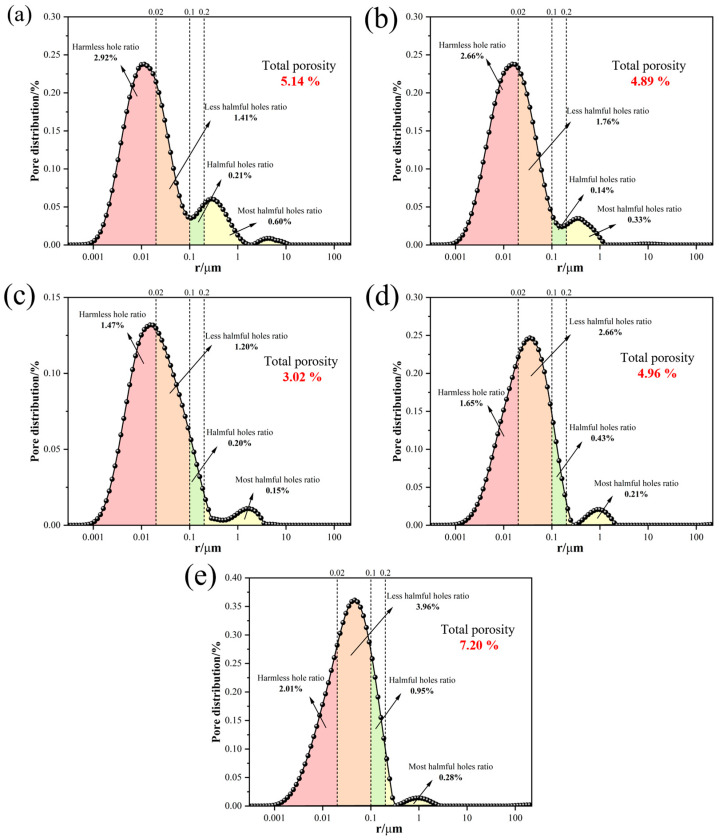
Pore characteristics of alumina cement under different curing temperatures: (**a**) 50 °C; (**b**) 60 °C; (**c**) 70 °C; (**d**) 80 °C; (**e**) 90 °C.

Figure 4 presents the pore structure characteristics of alumina cement at 24 h under different steam curing temperatures (50, 60, 70, 80, and 90 °C). The results indicate that the total porosity initially decreased and then increased with rising curing temperature. At 50 °C and 60 °C, the total porosity was 5.14% and 4.89%, respectively, with the hydration products consisting of the metastable hexagonal plate-like C_2_AH_8_ phase, resulting in relatively high porosity. When the temperature increased to 70 °C, the porosity reached its minimum value of 3.02%, which is ascribed to the transformation of hydration products into the granular stable C_3_AH_6_ phase, leading to a more compact structure and significantly reduced porosity. However, as the temperature further rose to 80 °C and 90 °C, although the hydration products remained as C_3_AH_6_, the excessively high curing temperature induced the formation of microcracks, causing the porosity to increase to 4.96% and 7.20%, respectively. To further clarify the influence of pore structure on performance, the pores in cementitious materials can be classified by size according to the criteria proposed by Zhongwei Wu [29]: more harmful (>200 nm), harmful (100–200 nm), less harmful (20–100 nm), harmless (<20 nm). The presence of more harmful and harmful pores has been shown to significantly weaken mechanical properties [30]. As shown in Figure 4, the proportions of these detrimental pores were the lowest at 70 °C, with more harmful pores at 0.20% and harmful pores at 0.15%, confirming that this temperature effectively suppresses the formation of harmful pores. In contrast, at 80 °C and 90 °C, the proportion of harmful pores increased to 0.43% and 0.95%, respectively, while that of more harmful pores rose to 0.21% and 0.28%. This trend aligns with the observed microcrack formation and the increase in total porosity, further confirming that excessively high curing temperatures degrade the pore structure.

The above results demonstrate that the steam curing temperature dictates the phase composition and microstructure of alumina cement by influencing the hydration pathway. Overall, a curing temperature of 70 °C achieves an optimal balance between phase transformation and microstructural densification. This temperature ensures the complete conversion of the metastable C_2_AH_8_ to the stable C_3_AH_6_, thereby eliminating subsequent issues related to volume instability and strength loss. Concurrently, it facilitates a more complete hydration reaction, resulting in the lowest porosity (3.02%) and a minimal proportion of harmful pores, which collectively contribute to a highly dense microstructure. In contrast, lower temperatures retain metastable phases and yield a looser structure, while higher temperatures, despite forming the stable phase, can reduce the hydration driving force and induce thermal stress, leading to microcracks and increased porosity. Therefore, 70 °C is identified as the critical curing temperature for simultaneously achieving stable phase formation and an optimised microstructure.

### 3.2. Investigation on the Properties of Alumina Cement Adhesive

In light of the substantiated findings that the hydration products at a 70 °C steam curing temperature are the stable phase C_3_AH_6_ and AH_3_, with the absence of metastable phases CAH_10_ or C_2_AH_8_, and that the cement paste porosity is comparatively low, this study adopted a rapid curing regimen of 70 °C steam curing for a duration of 2 h. The following materials were utilised: alumina cement as the cementitious material, artificial porcelain sand as aggregate, silica fume as mineral admixture, a cement-to-sand ratio of 3, and a water-to-cement ratio of 0.18 for the preparation of the alumina cement adhesive.

#### 3.2.1. Flexural and Compressive Strength

**Figure 5:**
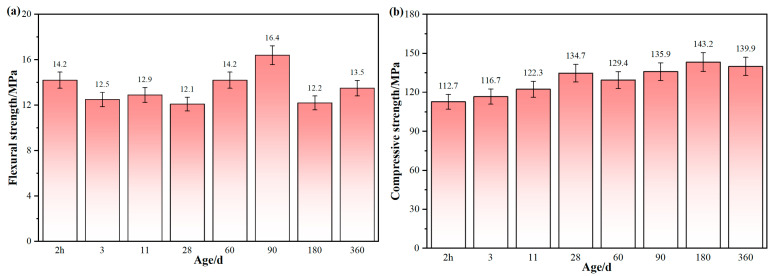
Mechanical properties of alumina cement adhesive at different ages: (**a**) Flexural strength and (**b**) Compressive strength.

Figure 5 illustrates the strength development pattern of the alumina cement adhesive produced through steam curing at 70 °C for 2 h over a 360-day curing period. Throughout this period, its flexural strength consistently exceeded 12 MPa. Compressive strength exhibited an upward trend that increased with age before gradually stabilising. As can be seen in Figure 5, after curing for only 2 h, the flexural strength and compressive strength reached 14.2 MPa and 112.7 MPa, respectively, already exceeding the industry standard requirements for high-strength adhesives (flexural strength ≥ 10.5 MPa, compressive strength ≥ 83.5 MPa) [31]. Compressive strength continued to increase over the next 28 days, peaking at 134.7 MPa on day 28. From days 60 to 180, the rate of strength gain slowed gradually but steadily, reaching a maximum of 143.2 MPa on day 180. After 360 days, the compressive strength was 139.9 MPa, indicating that it had essentially stabilised. It is noteworthy that the adhesive retained over 97% of its peak strength after 360 days, demonstrating significantly better long-term stability compared to many conventional CAC systems [16,32]. This demonstrates that alumina cement adhesive prepared using a 2 h rapid curing regimen at 70 °C exhibits high early strength and stable properties over the medium to long term.

#### 3.2.2. Dry Shrinkage Properties

**Figure 6:**
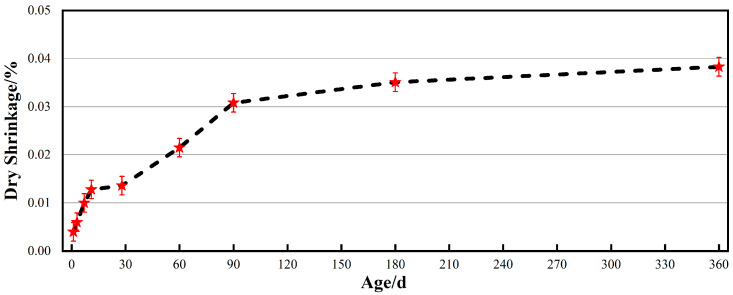
Drying shrinkage of alumina cement adhesive at different ages.

The dry shrinkage property is a key indicator of the dimensional stability of cementitious materials, particularly in applications requiring long-term reliability, such as ultra-high voltage insulators. A lower dry shrinkage rate helps maintain the integrity of the bond between the adhesive and porcelain or glass interfaces and components, ensuring the structural safety of the installation as a whole [33]. Figure 6 shows the time-dependent dry shrinkage curve of the alumina cement adhesive, which exhibits distinct phase characteristics. Within 90 days, the shrinkage rate increases rapidly, reaching approximately 0.0308% by day 90, representing 80% of the 360-day shrinkage value. This phase is primarily driven by the rapid evaporation of free water within the paste and the effects of capillary tension. From days 90 to 180, the dry shrinkage growth rate slowed significantly. By day 180, the shrinkage rate had reached approximately 0.0351%. During this period, shrinkage was primarily caused by moisture loss in finer capillaries and interlayer water migration within the gel. From days 180 to 360, the dry shrinkage rate reached complete stability, increasing only slightly from 0.0351% to 0.0383%. This demonstrates excellent long-term stability [34,35,36].

#### 3.2.3. Modulus of Elasticity

**Figure 7:**
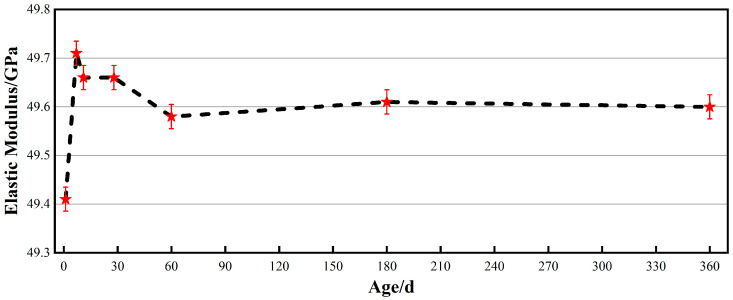
Elastic modulus of alumina cement adhesive at different ages.

The modulus of elasticity is a key mechanical parameter that influences the internal stress distribution of ultra-high voltage insulators. its long-term stability is of paramount importance [37]. As illustrated in Figure 7, the elastic modulus of the alumina cement adhesive initially increases and then stabilises over the 360-day ageing period. It consistently maintained a range between 49.3 and 49.8 GPa, stabilising at 49.6 GPa after 360 days. This indicates that the alumina cement adhesive prepared via 2 h steam curing at 70 °C completes primary hydration product formation and microstructural development in its early stages. A stable crystalline framework dominated by the cubic phase C_3_AH_6_ then forms internally. This structure effectively suppresses volume instability caused by later phase transformations and reduces shrinkage stresses induced by water migration through an optimised pore distribution. This inhibits microcrack propagation, giving the alumina cement adhesive outstanding long-term mechanical and volumetric stability.

### 3.3. Microstructural Study of Alumina Cement Adhesive

#### 3.3.1. Pore Characteristics

**Figure 8:**
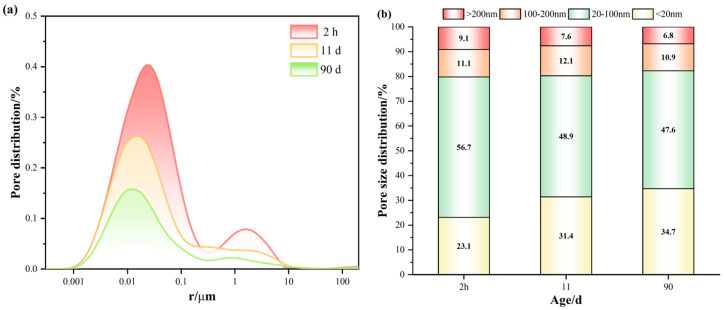
Pore characteristics of alumina cement adhesives at different ages (**a**) Pore size distribution curve; (**b**) Pore size distribution histogram.

Figure 8 shows how the distribution of pore sizes in an alumina cement adhesive change with age. As shown in Figure 8a, the characteristic pore size peak shifts progressively towards smaller diameters with increasing age of cure, indicating a gradual refinement of the internal pore structure [38]. Figure 8b further illustrates the evolution of the pore size distribution pattern: as the hydration process progresses, the proportion of gel pores below 20 nm increases significantly, while the proportion of medium-to-large pores above 20 nm decreases correspondingly. This reflects the continuous generation of hydration products that progressively fill the original pore spaces, driving the pore structure towards a finer scale [39]. Furthermore, quantitative porosity measurements corroborate this structural evolution; total porosity decreased markedly from 9.35% at one day to 6.22% at eleven days and declined further to 3.56% at ninety days. This pattern indicates sustained microstructural densification within the alumina cement adhesive during long-term curing, providing a crucial foundation for long-term macroscopic properties stability.

#### 3.3.2. Phase Composition

**Figure 9:**
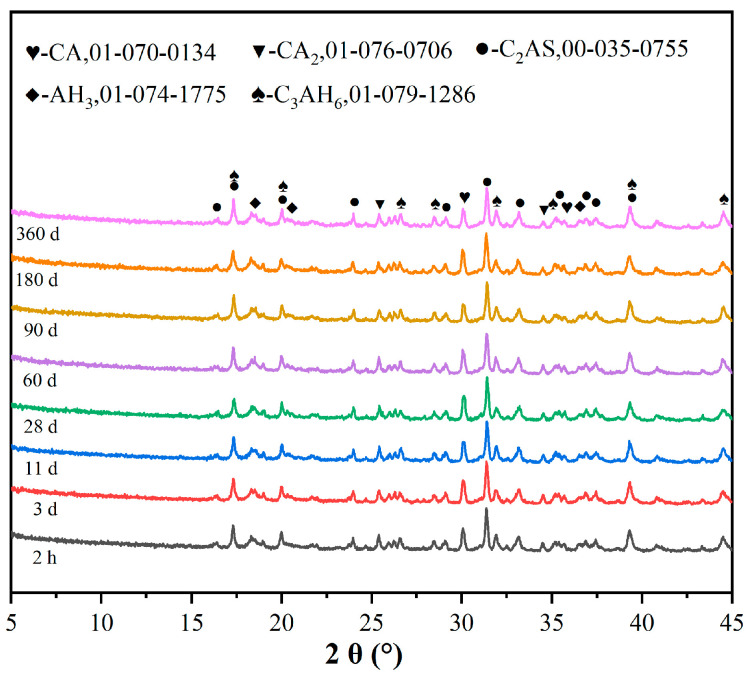
XRD patterns of alumina cement adhesive at different ages.

Figure 9 shows the X-ray diffraction patterns of the alumina cement adhesive prepared by steam curing at 70 °C for 2 h at various ages (1, 3, 11, 28, 60, 90, 180 and 360 days). The results indicate that the primary hydration products are C_3_AH_6_ and AH_3_, which exhibit distinct diffraction peaks at 2θ angles of approximately 17.3°, 31.9°, 39.2°, 44.4°, 18.3° and 20.3°, respectively. The peak shapes remain stable and their intensities show no significant variation with age. No metastable phases, such as CAH_10_ or C_2_AH_8_, were observed within the 360-day ageing period. This suggests that the alumina cement adhesive formed a stable phase composition during accelerated curing and that its crystal structure remained stable for at least 360 days. From a crystallographic perspective, this result confirms that the alumina cement adhesive exhibits excellent long-term phase stability.

#### 3.3.3. Microstructure

**Figure 10:**
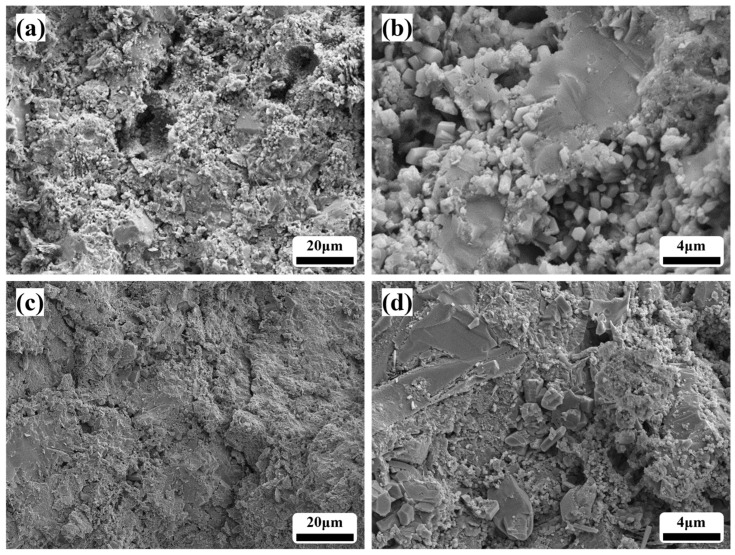
SEM images of the microstructure: (**a**,**b**) alumina cement paste; (**c**,**d**) alumina cement adhesive.

Figure 10 shows the microstructures of alumina cement paste and adhesive prepared by steam curing at 70 °C for 2 h. As Figure 10a,b illustrates, the hydration products in the alumina cement paste consist primarily of cubic C_3_AH_6_ crystals. These crystals exhibit relatively uniform sizes within the 1–3 μm range. Face-to-face inter-crystalline contacts form a continuous and stable spatial framework structure, indicating that the alumina cement hydration reaction proceeded fully under this curing regimen. The products crystallised well, forming a dense cement matrix. Figure 10c,d depict the microstructure of the alumina cement adhesive. The hydration products can be seen tightly enveloping the aggregate surfaces. C_3_AH_6_ and AH_3_ gels form an interwoven three-dimensional network between particles, which effectively fills interfacial voids and bridges aggregates with the cement matrix. This significantly improves the interfacial properties and load transfer efficiency of the cement adhesive system [40,41].

### 3.4. Thermomechanical Performance Testing of Alumina Cement-Bonded Porcelain Insulators

To evaluate the load-bearing capacity and reliability of alumina cement bonding agents in ultra-high voltage insulators, porcelain insulators were bonded using this material. Gradual electromechanical failure load tests were conducted on the bonded insulator specimens in accordance with the GB/T 1001.1-2021 standards. Specimens were deemed qualified only if they satisfied the criterion in Equation (5) throughout testing and exhibited no electrical faults, such as flashover or breakdown.

*X*–1.42*S* ≥ R (5)

where *X* is the average mechanical failure load of the specimen, *S* is the standard deviation, and R is the rated mechanical failure load of the specimen (550 kN).

First, a load equivalent to 80% of the specified electromechanical failure load was applied to the specimens. As shown in Table 2, the average electromechanical failure load for the bonded porcelain insulators was X = 671.63 kN, with a standard deviation of S = 4.63 kN. This satisfied the criteria of Equation (5), indicating that the alumina cement adhesive demonstrated excellent thermomechanical performance under an 80% load.

To further evaluate the feasibility of alumina cement-bonded porcelain insulators under extreme conditions, the applied load was increased to 100% of the specified electromechanical failure load. As shown in Table 2, under these severe conditions, the average electromechanical failure load was X = 696.69 kN, with a standard deviation of S = 2.12 kN. This satisfies the requirements of formula (5). The results of the two-stage load tests conclusively demonstrate that porcelain insulators bonded with the alumina cement adhesive developed in this study successfully passed the thermal–mechanical properties tests according to the GB/T 1001.1-2021 standard. This indicates their ability to withstand the combined effects of electromechanical loads and complex environmental conditions, providing robust experimental evidence for their use in ultra-high-voltage, heavy-duty insulators.

## 4. Conclusions

(1) The steam curing temperature has a significant influence on the phase composition and microstructure of the hydration products in alumina cement. At 20 °C, the hydration product is CAH_10_; at 50 or 60 °C, it is C_2_AH_8_; at 70 °C, the metastable phase disappears, yielding the stable phase C_3_AH_6_ with a porosity of just 3.02%. At 80 or 90 °C, the hydration product remains C_3_AH_6_, but microcracks develop, increasing the porosity to 5.59% and 5.14%, respectively.

(2) Alumina cement adhesive prepared via 2 h steam curing at 70 °C exhibits superior properties, with flexural and compressive strengths reaching 14.2 MPa and 112.7 MPa, respectively, within 2 h. Both flexural and compressive strengths exceed 12 MPa and 110 MPa within 360 days. Dry shrinkage remains below 0.04%, while the elastic modulus stabilises at around 49.6 GPa.

(3) The alumina cement adhesive prepared via accelerated curing exhibited stable hydration product phases over 360 days, with C_3_AH_6_ and AH_3_ as the primary crystalline phases. Pore volume gradually decreased with age, reaching 3.56% after 90 days. The microstructure was dense, with C_3_AH_6_ and AH_3_ gels tightly enveloping the surfaces of the aggregate and interweaving to form a compact, three-dimensional network.

## Figures and Tables

**Table 1:** Chemical composition of alumina cement (wt%).

SiO_2_	Al_2_O_3_	Fe_2_O_3_	CaO	MgO	TiO_2_	K_2_O	Na_2_O
7.37	48.14	1.91	36.38	0.46	2.21	0.64	0.07

**Table 2:** Thermal–Mechanical properties test results for insulators under 80% and 100% load.

Number	Electromechanical Destructive Load/kN	
	80% Load	100% Load
1	673.2 (J)	693.3 (J)
2	669.9 (J)	694.3 (J)
3	671.4 (J)	699.7 (J)
4	675.6 (J)	696.2 (J)
5	674.9 (J)	696.8 (J)
6	661.2 (J)	697.7 (J)
7	674.6 (J)	698.7 (J)
8	672.2 (J)	696.8 (J)
Average value X	671.63	696.69
Standard deviation S	4.63	2.12

## Data Availability

The original contributions presented in this study are included in the article. Further inquiries can be directed to the corresponding author.
